# Tuning of the magnetotransport properties of a spin-polarized 2D electron system using visible light

**DOI:** 10.1038/s41598-023-36957-w

**Published:** 2023-06-21

**Authors:** Maria D’Antuono, Yu Chen, Roberta Caruso, Benoit Jouault, Marco Salluzzo, Daniela Stornaiuolo

**Affiliations:** 1 Department of Physics, University of Naples Federico II, via Cinthia, 80126 Naples, Italy; 2 CNR-SPIN, via Cinthia, 80126 Naples, Italy; 3 Condensed Matter Physics and Materials Science Division, Brookhaven National Laboratory, Bldg. 480, P.O. Box 5000, Upton, NY 11973-5000 USA; 4 Laboratoire Charles Coulomb, UMR 5221, CNRS, Université de Montpellier, 34095 Montpellier, France

**Keywords:** Electronic properties and materials, Magnetic properties and materials, Two-dimensional materials, Surfaces, interfaces and thin films

## Abstract

We report on the effects of visible light on the low temperature electronic properties of the spin-polarized two dimensional electron system (2DES) formed at the interfaces between LaAlO$_{3}$, EuTiO$_{3}$ and (001) SrTiO$_{3}$. A strong, persistent modulation of both longitudinal and transverse conductivity was obtained using light emitting diodes (LEDs) with emissions at different wavelengths in the visible spectrum range. In particular, Hall effect data show that visible light induces a non-volatile electron filling of bands with mainly 3d$_{xz,yz}$ character, and at the same time an enhancement of the anomalous Hall effect associated to the magnetic properties of the system. Accordingly, a suppression of the weak-anti localization corrections to the magneto-conductance is found, which correlates with an enhancement of the spin-polarization and of the ferromagnetic character of 2DES. The results establish the LED-induced photo-doping as a viable route for the control of the ground state properties of artificial spin-polarized oxide 2DES.

## Introduction

Transition metal oxides show a wide range of properties making them excellent candidates for advanced electronic applications^1^. In particular, the heterostructure formed by a thin film of LaAlO$_3$ deposited on a SrTiO$_3$ substrate (LAO/STO) has gained great attention thanks to the several properties of the 2-dimensional electron system (2DES) developing at the interface, including superconductivity^2–4^ and Rashba spin-orbit coupling^5,6^. Due to the peculiar band structure and carrier density of the system, these properties are largely tunable using different stimuli, as for example magnetic or electric fields^7^. In recent years, several works have shown that oxide 2DES can also be manipulated using light with moderate/low power density and with wavelengths in the ultra-violet (UV), visible and near infrared (IR) ranges^8^. For example, light illumination can induce transition from weak localization to weak-antilocalization in LAO/STO magnetotransport^9^ and a modulation of the superconducting critical temperature^10^.

In this work we study the effect of light illumination on another functional property of atomic engineered 2DES, i.e. the spin-polarization, which can be induced by inserting few unit cells (u.c.) of EuTiO$_{3}$ (ETO) between LAO and STO. ETO is an antiferromagnetic band insulator, isostructural to STO, showing ferromagnetism below 8 K upon doping. In the LAO/ETO/STO heterostructure the 2DES charge carriers, above a critical value, start to fill Ti-3d bands with 3$d_{xz,yz}$ character, leading to the stabilization of a ferromagnetic order of Ti and Eu magnetic moments, and to a spin-polarization of the 2DES^11,12^. The data analysis presented in this work shows that such mechanism can be achieved not only using electric field effect^11–13^, but also using visible light. In the latter case, the stabilization of ferromagnetic correlations in the 2DES and the suppression of the weak-antilocalization corrections to the magneto-conductance are enhanced compared to field effect tuning. Furthermore, we show that, in some carrier density range, a switching-on of the spin-polarization of the system using only visible light stimuli is possible. The additional knob provided by visible light offers a unique opportunity to access unexplored regions of the phase diagram of oxide 2DES. Moreover, the ease of use of the illumination technique proposed in this work opens interesting opportunities for future applications.

## Results

The samples studied in this work are epitaxial LAO (10u.c.) /ETO (2u.c.)/STO (001) heterostructures deposited by pulsed laser deposition. The tuning of their transport properties was carried out using electric field effect in the back gate configuration combined with visible light irradiation provided by commercially available light emitting diodes (LEDs) mounted inside the cryostats, in the proximity of the samples’ surface. The use of commercial LED sources, while less selective in the illumination wavelength compared to laser sources^8^ (see Table 1 in the the “Methods” section) does not require an optical access for the cryostats, making this technique easier to implement.

In the next Sections we will firstly show that visible light induces a change in the LAO/ETO/STO 2DES sheet resistance, similarly to what has been observed in LAO/STO. Then we will focus on the effect of light on ferromagnetic coupling in LAO/ETO/STO by analyzing the Hall effect and the magnetoresistance data. Finally we will discuss the experimental results in view of the characteristic electronic structure of the 2DES and provide a simple model to explain the observed phenomena.

### Evolution of the resistance with the illumination time

**Figure 1:**
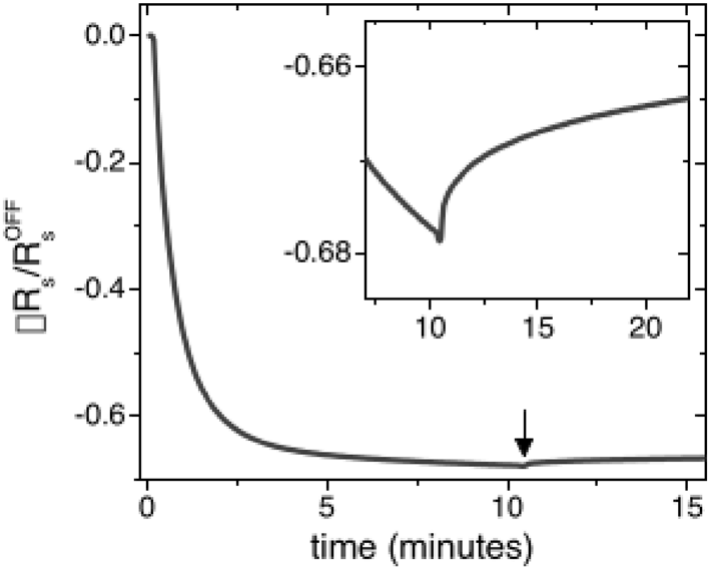
Time evolution of the sheet resistance $R_{s}$ of a LAO/ETO/STO Hall bar under visible light illumination. The inset shows the small recovery of $R_{s}$ when the LED is turned off. Measurements were performed at 5 K.

In Fig. 1 we present the typical evolution of the normalized sheet resistance $\Delta R_{s}=(R_{s}-R_{s}^{OFF})/R_{s}^{OFF}$ (where $R_{s}^{OFF}$ is the sheet resistance $R_{s}$ before illumination) of a LAO/ETO/STO Hall bar as a function of the illumination time at 5 K. A qualitatively similar behavior was observed for all wavelengths (listed in Table 1 in “Methods” section) and all gate voltages used. The zero of the horizontal axis corresponds to the instant when the LED source is switched on. The normalized sheet resistance $\Delta R_{s}$ under illumination shows an exponential-like decrease, with most of the variation taking place in the first 3 min of illumination. When the LED source is switched off, the low resistance state is retained, except for a few percent recovery, as reported in the inset of the figure. Similar trends have been revealed for several oxide 2DES based on STO under ultra violet and visible light illumination, typically performed using laser beams or halogen lamps^8,14–16^.

### Resistance versus temperature

**Figure 2:**
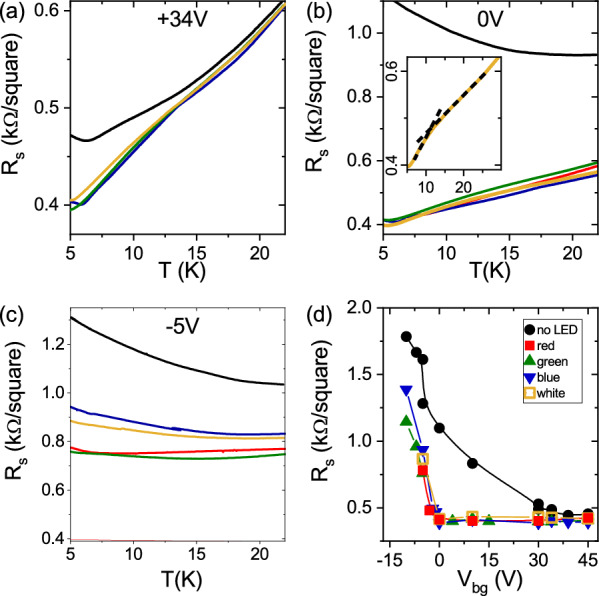
(**a**) to (**c**) Sheet resistance R$_{s}$ versus T for LAO/ETO/STO Hall bar measured at V$_{bg}$ = + 34 V, 0 V and − 5 V respectively. Black data were acquired in the absence of LED illumination, whereas red, blue and green data were acquired after illumination with LED of the corresponding color. Dark yellow data refer to illumination with white LED (see also legend in (**d**)). The inset of (**b**) shows a zoom of the curve obtained at V$_{bg}$ = 0 V after irradiation with white LED. The black dashed lines are a guide to the eye. All the measurements in these panels were performed keeping a constant cooling rate. (**d**) Shows R$_{s}$ as a function of the gate voltage after photodoping (T = 5 K).

Firstly, we analyze the evolution of the resistance as a function of the temperature under illumination. All the measurements reported in this section were performed following the same illumination protocol, similar to the one described in Ref.^9^: (i) cool down the sample to base temperature with a floating gate, (ii) apply a back gate voltage V$_{bg}$, (iii) illuminate the sample with LED light for 10 min, (iv) switch off the LED and wait 5 min for the resistance to stabilize before (v) starting the measurement ramping up the temperature at the rate of 1 K per minute. The feeding power to the LED source was set at the same value (25 mW) for all LED types and kept constant throughout the illumination time. At this power the change in the sample temperature, measured by a sensor in the proximity of the sample, is less than 0.5 K and is completely recovered as the LED is switched off. Panels (a) to (c) of Fig. 2 show the sheet resistance R$_{s}$ as a function of the temperature for several values of the back gate voltage V$_{bg}$, before illumination (black curves) and after illumination with red, green, blue and white LED lights (the latter data are represented in dark yellow). It is immediate to notice that for the positive values of V$_{bg}$, R$_{s}$ always drops, upon illumination, to the value of $\sim$400 $\Omega$ regardless of the gate voltage values and of the light color. On the other hand, for V$_{bg}<$0, the change in resistance upon illumination is substantially smaller and depends both on the gate voltage and on the light color. These observations are summarized in panel (d) of the same Figure.

Below 8 K, the LAO/ETO/STO 2DES shows ferromagnetism, tunable by electric field effect. A positive gate voltage shifts the Fermi level of the 2DES, increasing the carrier concentration and promoting conduction from Ti 3d$_{xz,yz}$ bands of the STO and ETO interfacial layers. The electrons in these bands can couple with the Eu spins within the ETO layers, thanks to their dispersion along the z-axis perpendicular to the interface, also within the STO unit cells, leading to a spin polarized 2DES^12^. A closer inspection to the data shown in panels (a) and (b) of Fig. 2 reveals that upon illumination with visible light, a change in the slope of the R vs. T curves appears at low temperature. In the inset of panel (b), for instance, we show the R$_{s}$ vs. T curve obtained at V$_{bg}$ = 0 V after irradiation with white light. A change in the slope in this curve is clearly visible, with a steeper decrease of the resistance below 10 K. This behavior is an indication of reduced scattering due to ferromagnetic coupling, as demonstrated by the combined analysis of magnetotransport properties and spectroscopy data reported in Ref.^11^. These observations are a first indication that visible light can be used to activate 3d$_{xz,yz}$ carriers, generating ferromagnetic coupling in the 2DES.

### Magnetotransport: anomalous Hall effect

**Figure 3:**
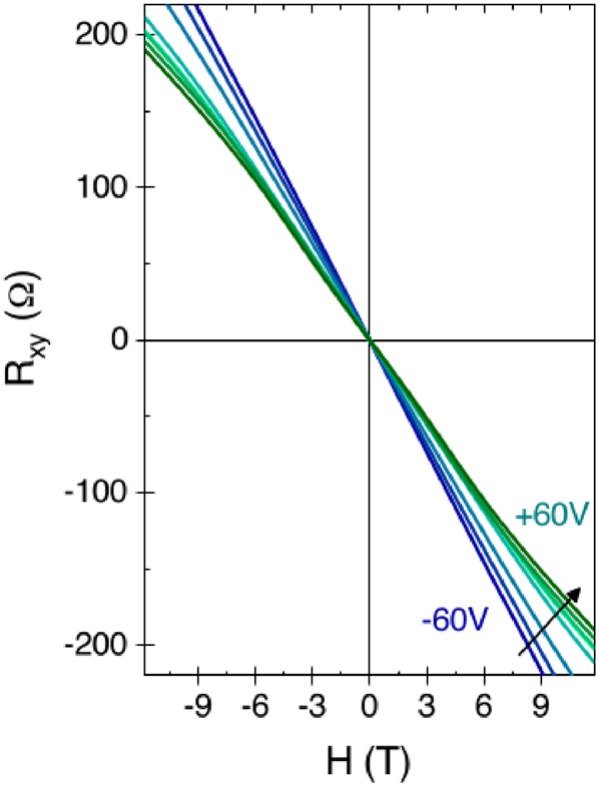
Hall effect measured at T = 2 K for different values of the gate voltage, from V$_{bg}$ = − 60 V(dark blue data) to V$_{bg}$ = + 60 V (dark green data).

Magnetic correlations and spin polarization in the 2DES are expected to induce direct effects in the magneto-transport. In particular due to the simultanous presence of Rashba-like spin-orbit coupling and carrier spin-polarization, the transverse Hall resistance is expected to develop an anomalous component proportional to the spin-polarization, giving rise to an anomalous hall effect (AHE). Conventional Hall effect consists in a build-up of a transverse electric field when a conductor lies in an external perpendicular magnetic field. In the case of a ferromagnetic conductor hosting spin-orbit coupling, the Hall resistance associated with the built-up electric field has an additional term, which depends directly on the magnetization of the material^17^. Figure 3 shows the transverse resistance R$_{xy}$ measured for LAO/ETO/STO for different values of V$_{bg}$ ranging from − 60 to + 60 V. At low values of the gate voltage, the R$_{xy}$ vs. H curves are linear. Increasing the gate voltage, two effects appear: an upward curvature at high field (H$>6$ T) due to the activation of multiband transport^18,19^ and a downward curvature at low field (H$<4$ T) due to the anomalous Hall component^11^.

In order to assess the effect of visible light on the anomalous Hall effect shown in Fig. 3, we switched on and off the LED between magnetic field runs to tune the sheet resistance progressively along the illumination curve of Fig. 1. The protocol used was the following: (i) cool the sample down to base temperature with a floating gate, (ii) apply the back gate voltage, (iii) switch on the LED for 2 min, (iv) switch off the LED and wait for the resistance to stabilize, (v) start the measurement ramping the magnetic field, (vi) at the end of the field sweep, switch again the LED for 2 min and repeat steps (iii) to (v). This protocol allowed us to obtain multiple measurements, corresponding to different values of the carrier concentration, at a fixed gate voltage.

**Figure 4:**
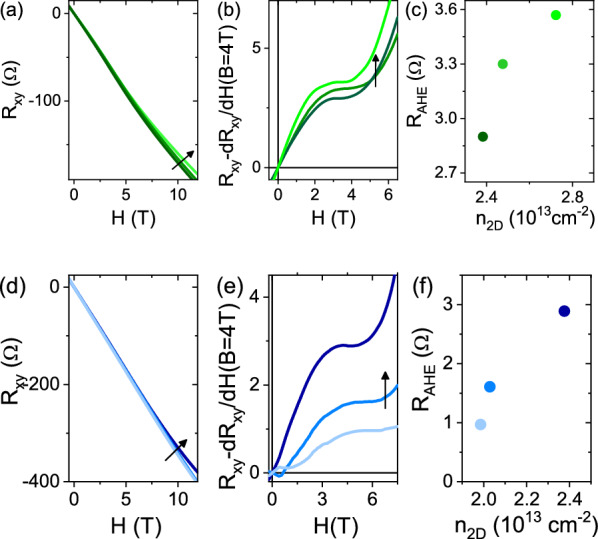
Hall effect measured at V$_{bg}$ = 60 V (**a**) and V$_{bg}$ = − 20 V (**d**) after repeated illumination steps. The black arrows indicate the direction of increasing illumination time. (**b**) and (**e**) show the data after subtraction of a linear component calculated around H = 4 T. These plots allow us to estimate the anomalous Hall component, defined as the saturation value of the curves at low field. (**c**) and (**f**) show the evolution of R$_{AHE}$ as a function of the carrier concentration n$_{2D}$ tuned by visible light.

In Fig. 4a,d we show Hall effect measurements obtained using the aforementioned protocol with V$_{bg}$ = + 60 V and − 20 V respectively. Each panel contains three curves measured after subsequent illumination steps with red (panel (a)) and blue (panel (d)) light. The arrows indicate the direction of increasing illumination time. In order to highlight the presence of an anomalous component in the transverse resistance, a linear component calculated around H = 4 T was subtracted from the original data (panels (b) and (e)). The anomalous Hall component R$_{AHE}$ corresponds to the plateau in the resulting curves^12^ (details on this procedure are reported in the “Methods” section). In panels (c) and (f) we plot the values of R$_{AHE}$ as a function of the carrier concentration n$_{2D}$ calculated from the slope of the R$_{xy}$ curves at high field (H$>6$ T). The data show that R$_{AHE}$ increases as the illumination time increases, following the same trend as the carrier concentration. This trend was revealed for all the back gate voltage values and visible light energies used.

**Figure 5:**
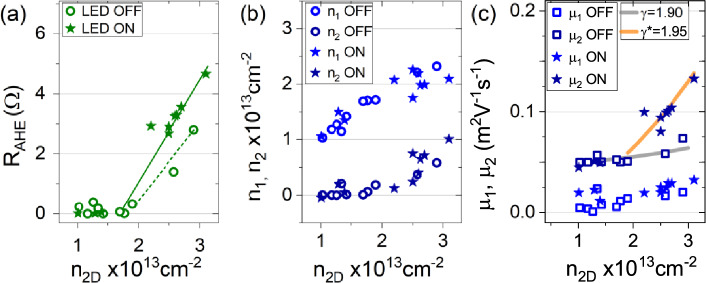
(**a**) Reports the R$_{AHE}$ values extracted from the anomalous part of the R$_{xy}$ curves modulated using only electric field effect (open dots) or electric field effect plus visible light irradiation (stars). (**b**) and (**c**) show the carrier concentration and mobility respectively extracted from two-band fit of the Hall data. The open symbols refer to gate-modulated transport while the plain stars to light-modulated one. n$_{1}$ ($\mu _{1}$) and n$_{2}$ ($\mu _{2}$) refer to the concentration (mobility) of carriers having d$_{xy}$ and d$_{xz,yz}$ orbital character respectively. The full lines in (**c**) refer to the function $\mu _{2}\propto n_{tot}^{\gamma }$.

The anomalous component due to AHE shown in Fig. 4b,e can be subtracted from the original R$_{xy}$ data, resulting in curves showing only the classical Hall effect contribution (see “Methods”). The fit of the latter using a two-band model allows us to extract the evolution of the carrier concentrations n$_1$ and n$_2$ (with n$_1$+n$_2$ = n$_{2D}$) and the mobility $\mu _1$ and $\mu _2$ of the two bands involved in the transport. The subscripts 1 and 2 refer to carriers having d$_{xy}$ and d$_{xz,yz}$ orbital character respectively. Figure 5 shows the result of this fit applied to the data for which the carrier concentration n$_{2D}$ was tuned using field effect and light. Panel (a) shows the values of R$_{AHE}$ tuned using field effect (open dots) or light (stars), while panels (b) and (c) show the values of carrier concentration and of mobility attributed to the two types of carriers in the 2DES. The values of R$_{AHE}$ obtained with light are larger than those obtained with field effect only [panel (a)]. However, field effect and visible light irradiation lead to similar maximum values of n$_{2}$, which correspond to d$_{xz,yz}$ type carriers having higher mobility, [panel (b)]. The d$_{xz,yz}$ carriers are those responsible for ferromagnetic coupling in LAO/ETO/STO^12^; the fact that light and field effect lead to a similar increment of these carrier concentration (n$_{2}$) indicates that the enhancement of ferromagnetic effect suggested by the change in the R$_{s}$ vs T slope of Fig. 2a,b and by the high values of R$_{AHE}$ of Fig. 5a in the presence of light illumination is not simply linked to the 3d$_{xz,yz}$ carrier concentration, but also to other effects. In panel (c) we show the mobility data extracted by the two-band fit. The mobility of 3d$_{xz,yz}$ carriers increases by positive gate voltages and by light illumination, but light-induced carriers are substantially more mobile (star scatter point), compared to gate-induced carriers (open circles). Thus, the two-band fit results, combined with the evolution of the R$_{AHE}$ parameter, suggest that the visible light irradiation excites d$_{xz,yz}$ carriers with higher mobility, possibly more effective in mediating FM coupling (see also Fig. S1 in Supplementary Information for additional data).

### Magnetotransport: weak anti-localization

**Figure 6:**
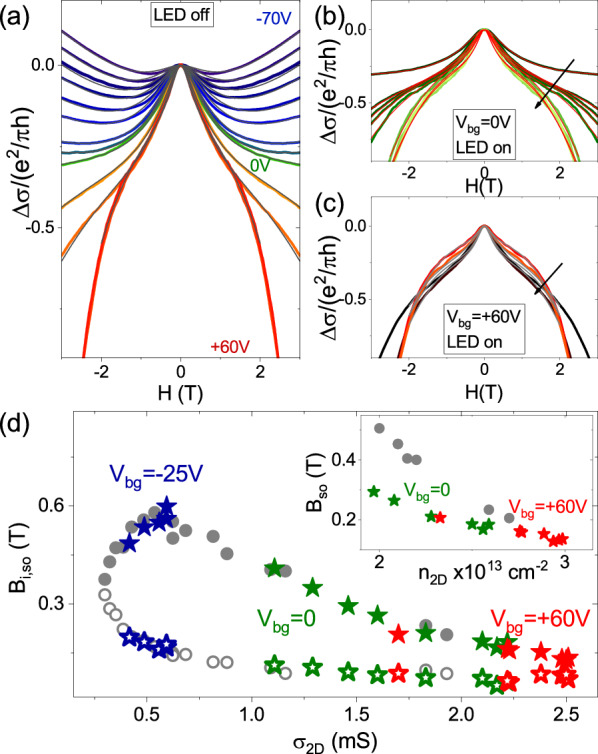
(**a**) Normalized differential magnetoconductance $\Delta \sigma$ of LAO/ETO/STO heterostructure as a function of the gate voltage measured at T = 2 K. (**b**) and (**c**) show data measured at fixed gate voltage V$_{bg}$ = 0 V and + 60 V respectively after subsequent illumination steps. The arrows departing from the LED OFF curve, indicate the direction of increasing illumination time. (**d**) Shows the inelastic B$_i$ (open symbols) and spin-orbit B$_{so}$ (filled symbols) fields extracted by fitting the magnetoconductance curves. The grey dots refer to gate modulated transport while the stars refer to light modulated transport at V$_{bg}$ = − 25 V (blue), 0 V (green) and + 60 V (red). In the inset of (**d**) the B$_{so}$ field is plot as a function of the total carrier concentration n$_{2D}$.

Another indication of visible light-enhanced magnetic correlations in the 2DES comes from the analysis of the magnetoconductance data. In oxide 2DES magnetoconductance measurements can show signatures of weak anti-localization (WAL) due to spin-orbit coupling, and a transition from WAL to weak localization (WL) with decreasing carrier concentration, as reported for LAO/STO 2DES^5,6,20^. LAO/ETO/STO 2DES shows WAL corrections to the magnetoconductance qualitatively similar to that of the LAO/STO system^11^. However, the evolution of WAL corrections with carrier density in LAO/ETO/STO is affected by the insurgence of FM correlations. Above the Lifshitz transition, the WAL corrections and the ferromagnetism of the 2DES compete, as it is well known that the quantum corrections to the magnetoconductance are suppressed in ferromagnetic films^13^. In Fig. 6a we plot the differential magnetoconductance $\Delta \sigma$ curves as function of the gate voltage without light irradiation (LED off), while in Fig. 6b,c we show data acquired with successive light irradiation steps, following the same procedure described in the previous paragraph for the Hall effect measurements, at V$_{bg}$ = 0 and + 60 V respectively. While a theoretical magneto-conductance model suitable for the intermediate regime of spin-orbit coupling and magnetic splitting which applies to our system is currently missing, we can in any cases quantify the effects of the gate and of the light illumination resorting to non-magnetic models, such as the Maekawa and Fukuyama (MF) formula, which gives good results when applied to LAO/STO^5,6^ and LAO/ETO/STO^13^. In Fig. 6a–c we show the fitting results as gray and red lines. In Fig. 6d we show the sheet conductance dependence of the inelastic B$_{i}$ (open symbols) and spin-orbit B$_{so}$ (filled symbols) fields as obtained from the fit. The data obtained as function of the gate voltage are shown as grey scatter points, while the data after visible light irradiation are shown as blue, green and red scatter points for V$_{bg}$ = − 25 V, 0 and + 60 V respectively. For all data shown, B$_{i}$ initially decreases and B$_{so}$ increases with increasing sheet conductance, similarly to what happens in LAO/STO. Around $\sigma ^{0}_{2D}$ = 0.7 mS, B$_{so}$ reaches a maximum and then starts to decrease. This behavior is due to the activation of ferromagnetic coupling for n$_{2D}^c> 2x10^{13}$ (corresponding to $\sigma ^{0}_{2D}>$0.7 mS) which masks the WAL corrections to the magnetoconductance^13^.

The data under illumination trace nicely those obtained by changing the gate voltage, including the non-monotonic behavior of B$_{so}$ due to the onset of ferromagnetic transition, which in this case is induced by light.

Light irradiation, however, allows us to reach higher conductance values, compared with gate tuning in agreement with data shown in Fig. 2d). In the inset of panel (d) the spin orbit field B$_{so}$ is plot as a function of the total carrier concentration n$_{2D}$. The plot shows that the B$_{so}$ values obtained from the data after visible light irradiation are consistently lower than those obtained using field effect gating only, indicating stronger ferromagnetic coupling after irradiation with visible light, in agreement with the conclusions drawn from the analysis of Hall effect (Fig. 5).

## Discussion

The main findings reported in the previous Sections are summarized below:

- at low temperature, photo-doping induces persistent changes in the sheet conductance and carrier density (Fig. 1).
- At V$_{bg}\ge$0, photo-doping leads to a reduction of the 2DES sheet resistance to values lower than those achievable by using electric field effect only (Fig. 2).
- At V$_{bg}<$0, the 2DES sheet resistance values achieved using photo-doping are substantially higher than the minimum possible, and depend on the peak wavelength of the LED used (at fixed power and irradiation time) (Fig. 2).
- Above the carrier density threshold corresponding to the Lifshitz transition, we do see evidence of AHE and of magnetic correlations in the 2DES, matching with the appearance of carriers with highest mobility, i.e. 3d$_{xz,yz}$ carriers (Figs. 4 and 5). The mobility vs. carrier density analysis suggests a larger enhancement of the mobility by light irradiation compared to back-gating and, at the same time, an increase of the anomalous Hall resistance (Fig. 5).
- Enhanced magnetic correlations are confirmed by the analysis of the WAL corrections to the magneto conductance. The inset of Fig. 6d demonstrates that when a given carrier concentration is reached using light irradiation, the WAL corrections to the magnetoconductance are suppressed, due to ferromagnetic coupling, more than what happens when the same carrier concentration is reached using field effect tuning only.

**Figure 7:**
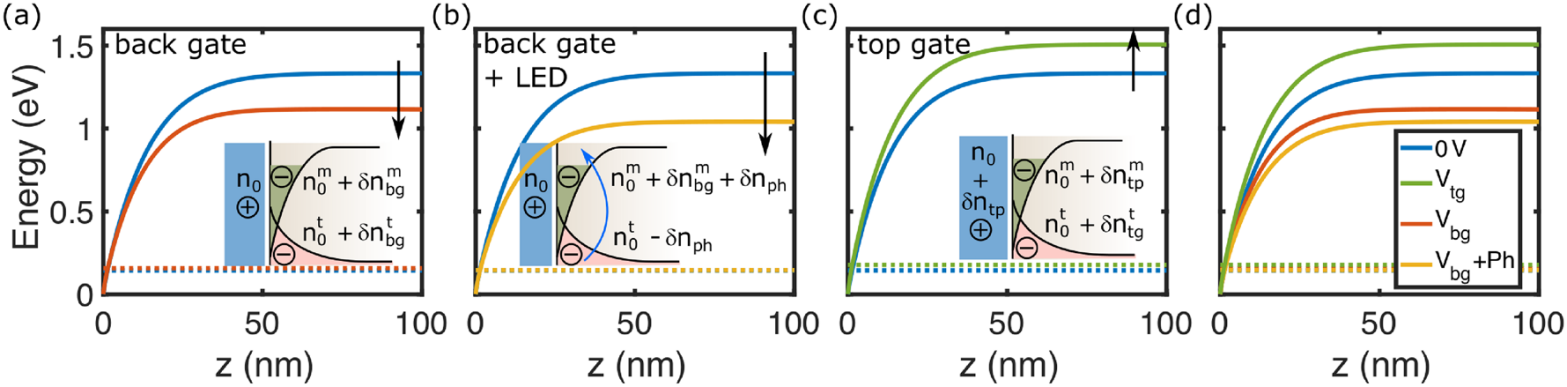
Schrödinger-Poisson numerical simulations of the quantum well tuned using: (**a**) V$_{bg}$ = 60 V (red line), (**b**) V$_{bg}$ = 16 V plus photo-doping (yellow line), (**c**) top gate V$_{tg}$ = 0.64 V (green line) and (**d**) comparison of all the cases. The blue solid line indicate the initial state. The dotted lines indicate the Fermi levels and the z-axis indicate the distance from the ETO/STO interface. In (**a**)–(**c**) the gate values were chosen in order to add $0.9 \times 10^{13} \, \text{cm}^{-2}$ mobile electrons into the interface. The sketches in the insets show that the density of positive charges $n_0$ is unchanged in the back gating (inset of (**a**)), whereas it amounts to $n_0 + \delta n_{tp}$ in top gating^25^ (inset of (**c**)). Both gates induce mobile and trapped charges^26^, we assume $\delta n_{bg/tg}^t = 0.55 \times 10^{13} \, \text{cm}^{-2}$, while there are only mobile carriers after illumination, which excites $\delta n_{ph}$ electrons from trapped defects to interface (**b**).

In order to understand in more details the combined effect of back-gating and photo-induced carriers, we introduce a qualitative model to account for the inherent differences between the two methods of carrier-tuning, as discussed previously in the LAO/STO case^21,22^. We assume that the total electron density of the 2DES is composed of mobile carriers and of a fraction of localized carriers which presumably are mostly present at the interface and/or in the ETO layer. By solving self-consistently Schrödinger-Poisson (SP) equations (in analogy with Ref.^23–26^), we can determine the distribution of carriers and the effect of both electrostatic gating and light on the confining potential (see also Fig. S2 of Supplementary Information). For completeness, the simulations were performed also in the top gate configuration (although this was not used in our experiments). In Fig. 7 we compare back-gating, top-gating and photo-doping on the 2DES confining potential. In these calculations we used values of back and top gating and photo-doping leading to a similar amount of accumulated carriers.

Top and back gating act in different ways on the confining potential of the 2DES: increasing back gate repels the electrons away from the interface, thus substantially deconfining the 2DES inside the STO crystal, whereas increasing top gate sharpens the confining potential, pushing the carriers towards the interface and raising the Fermi level, as schematically shown in Panels (a) and (c) of Fig. 7. In the case of LAO/STO 2DES, it was found that, as a consequence of this mechanism, the mobility of the carriers increases faster when the carrier concentration is tuned using back gate, compared to what happens with top gating, as STO substrate is less disordered than the interface. In particular, the relation between the mobility of 3d$_{xz,yz}$ carriers $\mu _{2}$ and the total carrier density n$_{tot}$ tuned by the two methods of gating, can be reproduced by the empirical relation $\mu _{2}\propto n_{tot}^{\gamma }$, where $\gamma$ = 1.90 for back gating and $\approx$ 1 for top gating^22^. In LAO/ETO/STO 2DES, we find $\gamma$ = 1.9 for back gating (grey line in in Fig. 5c), similarly to what reported for LAO/STO. The effect of photoactivation on the confining potential is, on the other hand, shown in Fig. 7b. In this case, the confining potential is tuned through photo-excitation of a fraction of trapped electrons, which are retained at the interface after illumination, resulting in a more pronounced deconfinement of the electrical potential. As a consequence, photo-doping is even more efficient than back-gating in creating higher mobility carriers, as confirmed by the parameter $\gamma ^{*}\approx$1.95 found for our data under light illumination (orange line in Fig. 5c). It should be noted that this phenomenological model does not consider the possibility that the increase of mobility is also due to the reduction of elastic and inelastic scattering due to the enhanced spin-polarization of carriers, a scenario which probably conspires together with the one discussed above in explaining the experimental results.

The above picture also explains why the photo-doping is non volatile, as shown Fig. 1: when the light is turned off, the carriers closer to the interface recombine and get trapped again, resulting in the small recovery of the resistance shown in the inset of the same Figure; whereas those deeper in the STO, where fewer impurities are present, are separated from the trapping centers, leading to a persistent photo-doping effect. Moreover, when a negative back gate voltage is applied, the confining potential shrinks and less carriers become available for photo-doping (see Fig. 2c,d), therefore the minimum resistance obtained by photo-doping is larger than the lowest possible value, which is always reached with V$_{bg}\ge$0.

This model captures well the overall mechanism of persistent photo-doping by light-irradiation in oxide 2DES, like (001) LAO/STO and LAO/ETO/STO. However, it remains to be discussed the origin of trapped carriers and, in particular, their energetics on the basis of our knowledge of the electronic structure of the LAO/ETO/STO system. Furthermore, the mechanism leading to an overall enhancement of the anomalous Hall effect and of magnetic correlations in the 2DES by light irradiation remains to be explained. The energies of the visible radiations used in the experiments described in this work (see Table 1 in “Methods”) lie well below the STO band gap (3.2 eV); therefore, a light-induced direct promotion from the valence band to the conduction band of STO can be excluded. In LAO/STO, sub-gap photoactivation has been explained taking into account the presence of in-gap states 1.3 eV below the conduction band, originating from impurities and/or oxygen vacancies^9,12^. For instance, angle integrated resonant photoemission spectroscopy (RESPES) data clearly show in-gap states in STO and oxygen deficient LAO/STO^12^. In the case of LAO/ETO/STO, on the other hand, RESPES measurements reveal a strong Eu4f peak around − 1.95 eV and a hybridization of Eu4f, Eu5d, and O2p states^12^. Together with density functional theory calculations, these results indicate that the Eu4f, Eu5d, and O2p states are also spin-polarized^12^ (see also Fig. S3 of Supplementary Information). Thus, electrons photo-excited from these bands, could be spin-polarized.

As matter of fact, the highest values of the AHE resistance, and thus degree of magnetic correlations, are observed in the case of light irradiation. This effect could be related to excitation of spin-polarized carriers at the interface, which distribute over the 2DES thickness well inside the STO. It is worth noting that if the carrier induced by photodoping would not be spin-polarized, we should expect an overall decrease of the Anomalous Hall effect, and not an enhancement.

Thus, while the data and the analysis suggest that in LAO/ETO/STO the photodoping mechanism is similar to the LAO/STO case, the presence of spin-polarized bands in the ETO and at the interface with the STO might contribute to the photo-excitation of spin-polarized electrons into the conduction band even using simple LED sources (without the need of circular polarized light).

In conclusions, in this work we show how commercial LED sources can be used to control the transport properties in LAO/ETO/STO. Reduced scattering to due FM coupling mediated by light-activated carriers is visible in the R vs.T curves. A deeper analysis of the Hall effect allows us to conclude that such carriers possess an higher mobility compared to those activated by field effect. Moreover, there are indications that the higher mobility, together with a possible intrinsic spin-polarization of these photo-excited carriers, conspire in mediating the FM coupling and promoting AHE. Therefore, the combined use of visible light and gate voltage enables us to reach regions of the the LAO/ETO/STO 2DES phase diagram which are not accessible using field effect tuning alone.

## Methods

### Samples preparation and measurement set-up

The samples used in this work are epitaxial LAO (10u.c.) /ETO (2 u.c.)/STO (001) heterostructures deposited by pulsed laser deposition. A KrF excimer laser (wavelength: 248 nm) was focused on sintered Eu$_2$Ti$_2$O$_7$ and crystalline LAO targets. The thin films growth was monitored using Reflection High-Energy Electron Diffraction (RHEED). During the deposition the TiO$_2$ terminated STO substrate is kept at 680 $^\circ$C in an oxygen partial pressure of 1x10$^{-4}$ mbar. Following deposition, the samples are slowly cooled down to room temperature in the same oxygen pressure. A detailed investigation of the structural properties of LAO/ETO/STO heterostructures has been reported in Ref.^11^, revealing high structural and chemical order. Some of the samples investigated were patterned to realize a Hall bars 500 μm wide, using photolithography and low-temperature ion milling^27^.

The samples were measured using a variable temperature cryostat (base temperature: 5 K) and a flow cryostat (base temperature: 2 K), the latter equipped with a superconducting coil (maximum magnetic field: 12T). The tuning of the transport properties was carried out using electric field effect in the back gate configuration: a metal layer was deposited on the back of the STO substrate and electric field was applied between the 2DES and this counter-electrode. The response to visible light was analyzed by using commercially available LEDs mounted inside the cryostats and in proximity to the samples’ surface. The LEDs light peak wavelengths $\lambda$ are listed in Table 1. We point out that the emission spectrum of the LED sources, as declared by the manufacturers, includes some dispersion around the dominant wavelength. In Table 1 we report this information as spectral line half width ($1/2 \Delta \lambda$). Therefore the covered range of wavelengths include photons from the near-IR to the near-UV range.

**Table 1:** LED lights properties.

LED light color	$\lambda$ (nm)	Energy (eV)	$(1/2 )\Delta \lambda$ (nm)
Red	655	1.89	20
Green	525	2.36	15
Blue	470	2.63	10
White	From 450 to 650	From 2.75 to 1.9	

Blue, green and white LEDs were purchased from Nichia company (models NSPB300B, NSPG310B, NSPW300DS respectively), red LEDs were purchased from Kingbright (model L-7104SRC-D). These LEDs were selected in order to have a similar feeding power (see main text).

### Fitting procedure of the transverse resistance R$_{xy}$ data

**Figure 8:**
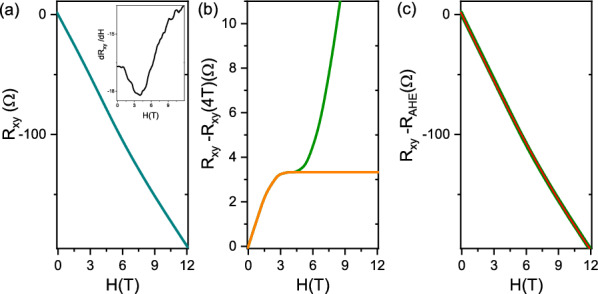
Fitting procedure of R$_{xy}$. (**a**): R$_{xy}$ raw data obtained for V$_{bg}$ = + 40V and derivative (inset). Panel (b): curve obtained by subtracting a linear slope (green data) and extrapolation of such curve to the maximum field value (orange curve). Curve obtained by subtracting the orange data of (**b**) to the raw R$_{xy}$ data (green curve) compared with the two band fit (red curve).

In this section we report the procedure used to extract the transport parameters reported in Figs. 4 and 5. Panel (a) of Fig. 8 shows typical R$_{xy}$ raw data at high carrier concentration (n$_{2D}^c>2\times$10$^{13}$ cm$^{-2}$). The data exhibit a low field downward curvature and an high field upward one (as highlighted by the derivative shown in the inset) due to two contributions to the Hall effect, namely the anomalous Hall effect and the classical multiband transport. In order to analyze the two different contributions, we need to separate them from the raw data. Firstly, we subtracted from the R$_{xy}$ data a linear component corresponding to its slope at 4 T (i.e. the minimum of the dR$_{xy}$/dH curve). The result is shown in panel (b) (green curve). The plateau of this curve represents the value of the anomalous component R$_{AHE}$ of the transverse resistance reported in Figs. 4 and 5 of the main text.

The anomalous component was then subtracted from the data. We firstly extrapolated this component to the maximum field value (orange curve in panel 8b), then subtracted from the raw R$_{xy}$ data. The resulting curve, shown in green in panel 8c, contains only the classical Hall contribution and can be fit using a two-band model^12^:

1 $$\begin{aligned} R_{xy}(B)=-\frac{B}{e}\frac{\frac{n_{1}\mu _{1}^{2}}{1+\mu _{1}^{2}B^{2}}+\frac{n_{2}\mu _{2}^{2}}{1+\mu _{2}^{2}B^{2}}}{\left( \frac{n_{1}\mu _{1}}{1+\mu _{1}^{2}B^{2}}+ \frac{n_{2}\mu _{2}}{1+\mu _{2}^{2}B^{2}}\right) ^{2}+\left( \frac{n_{1}\mu _{1}^{2}}{1+\mu _{1}^{2}B^{2}} +\frac{n_{2}\mu _{2}^{2}}{1+\mu _{2}^{2}B^{2}} \right) B^{2}} \end{aligned}$$

where n$_{1}$, $\mu _{1}$, n$_{2}$, $\mu _{2}$ are the carrier densities and the mobilities of the two bands, and B = $\mu _{0}$H. For the fitting, we used the constraint: 1/R$_{s}$ = e(n$_{1}\mu _{1}$+n$_{2}\mu _{2}$) where R$_{s}$ is the sheet resistance.

### Magnetoconductance fit

The differential magnetoconductance curves of Fig. 6 were fit using the MF formula including a parabolic classical background:

2 $$\begin{aligned} \begin{aligned} \frac{\Delta \sigma (B)}{\sigma _0}=&\Psi \left( \frac{B}{B_{i}+B_{so}}\right) +\frac{1}{2\sqrt{1-\gamma ^2}}\Psi \left( \frac{B}{B_{i}+B_{so}\left( 1+\sqrt{1-\gamma ^2}\right) }\right) +\\&-\frac{1}{2\sqrt{1-\gamma ^2}}\Psi \left( \frac{B}{B_{i}+B_{so}\left( 1-\sqrt{1-\gamma ^2}\right) }\right) +A_{k}\frac{\sigma ^{0}_{2D}}{\sigma _{0}}\frac{(\mu B)^2}{1+A_k (\mu B)^2} \end{aligned} \end{aligned}$$

where $\sigma ^{0}_{2D}$ is the sheet conductance at zero magnetic field, $\sigma _{0}= e^{2}/\pi h$, $\Psi (x)=ln(x)+\psi (\frac{1}{2}+\frac{1}{x})$ (with $\psi$ the digamma-function), $\gamma$= g$\mu _B$B/4eDB$_{so}$ (with $\mu _B$ the Bohr magneton and D the 2DES diffusivity), and $\mu$ is the 2DES mobility. The constant $A_k$ gives account of the strength of the classical parabolic term.

### Schrödinger-Poisson numerical simulations of the quantum well

The confining potential, energies and wave functions can be evaluated using the Schrödinger-Poisson equations based on effective mass approximation^23–26,28^:

3 $$\begin{aligned}{} & {} \left[ \frac{\hbar ^2}{2 m_\alpha ^z} \frac{d^2}{d z^2}+e \phi (z)+\varepsilon _{i \alpha }\right] \zeta _{i \alpha }(z)=0, \quad i=1,2,3, \ldots \end{aligned}$$

4 $$\begin{aligned}{} & {} -{\varepsilon _0} \frac{d}{d z}\left[ \varepsilon _r(E) \frac{d}{d z} \phi (z)\right] =\rho (z) \end{aligned}$$

where $\zeta _{i \alpha }(z)$ is an envelope wave function, the index $\alpha = xy,yz,xz$ represents the three Ti $t_{2g}$ orbitals, $d_{xy},d_{yz},d_{xz}$. The effective masses of the various bands $m_\alpha ^z$ are extracted from ARPES data^12^: $m_l = 0.4\,m_e$ for $m_{xz}^z$ and $m_{yz}^z$ while $m_h = 10\,m_e$ for $m_{xy}^z$. There are $n_0$ positive countercharges in LAO layer to keep the system neutral^25^, therefore the electric field satisfies the condition:

5 $$\begin{aligned} \varepsilon _r(0^+) E(0^+) = {\left\{ \begin{array}{ll} -\dfrac{en_0}{\varepsilon _0}, &{} \text {for back gating} \\ -\dfrac{e(n_0+\delta n_{tp})}{\varepsilon _0}, &{} \text {for top gating} \end{array}\right. } \end{aligned}$$

where $\varepsilon _0$ is the vacuum permittivity. The ETO/STO region is noted as $[0^+, L]$, in this region the density of the total electronic charges $n^{tot}$ includes the mobile and trapped electrons, which equal the positive charges in the LAO layer $n_0$. The charge in ETO/STO layers can be altered by electric field or illumination. As shown in Fig. 7, the top/back gating modifies the carrier density of the mobile charges by reshaping the confining potential, while photo-doping is assumed to increase mobile electrons via excitation from trapped centers:

6 $$\begin{aligned} n_0 = {\left\{ \begin{array}{ll} \left( n_0^m + \delta n_{g}^m\right) + \left( n_0^t+\delta n_{g}^t\right) , &{} \text {for gating}\\ \left( n_0^m + \delta n_{g}^m + \delta n_{ph}\right) + \left( n_0^t-\delta n_{ph}\right) , &{} \text {for photo-doping plus gating} \end{array}\right. } \end{aligned}$$

where the density of the initial mobile electrons is assumed to be $n_0^m = 1.9 \times 10^{13}\, \text{cm}^{-2}$ in agreement with our experiments. We assume the distribution of the initial trapped electrons $N_0^t (z) = \frac{n_{t0}}{\lambda }e^{-\frac{z}{\lambda }}$, here $\lambda = 50 \, \text{nm}$^26^ and $n_{t0} = 1.41\times 10^{14}\, \text{cm}^{-2}$, $n_0^t = \int dz N_0^t (z)$. When a back gate of $60\, \text{V}$ is applied in experiments (or a top gate of $0.64\, \text{V}$ in calculation), the mobile charges density is increased by $\delta n_{g}^m = 0.9 \times 10^{13}\, \text{cm}^{-2}$ and the trapped charge density induced by gate is assumed to be $\delta n_{g}^t = 0.55 \times 10^{13}\, \text{cm}^{-2}$. In order to get the same amount of mobile charge variation in the case that both back gate and photo-doping ara applied to the system, it is supposed that a back gate of $16\, V$ is used and photocarriers $\delta n_{ph} = 0.46 \times 10^{13}\, \text{cm}^{-2}$ are excited from the trapped centers to the interface. The amount of electrons $\delta n_g$ introduced by top/back gating, can be evaluated using a parallel plates capacitor model:

7 $$\begin{aligned} \delta n_g = \int _{0}^{V_g} \dfrac{\varepsilon _0}{ed_{ST0/LAO}}\varepsilon _r^{STO/LAO}dV_g \end{aligned}$$

The LAO permittivity $\varepsilon _r^{LAO} = 20$ is constant^29^, while that of the STO substrate is field dependent^24^:

8 $$\begin{aligned} \varepsilon _r^{STO}(E) = 1 + \dfrac{B}{[1+(E/E_0)^2]^{1/3}} \end{aligned}$$

where $B = 25462$, $E_0 = 82213 \, V/m$ and $E = V_{bg}/d_{STO}$. A loop of Schrödinger-Poisson self-consistency was performed through the following iterative steps:

- Using a triangular-potential approximation, Airy equation is taken as a trivial wavefunction^28,30^ $\zeta _0(z) = \sqrt{\dfrac{1}{b^3}}ze^{-\frac{bz}{2}}$ (with $b = [\frac{33\pi }{2}(n_0^m + n_g)a_B^2]^\frac{1}{3}\frac{1}{a_B}$ where Bohr radius $a_B = \dfrac{4\pi \varepsilon _r\varepsilon _0*\hbar ^2}{m^*e^2}$) to calculate the mobile charge distribution:
  9 $$\begin{aligned} \rho ^m(z) = -e(n_0^m + \delta n_g^m)|\zeta _0(z)|^2 \end{aligned}$$
  The trapped charges distribution can be described as $\rho _0^t(z) = -e N_0^t(z)$. The total charge distribution is given as:
  10 $$\begin{aligned} \rho (z) = \rho ^m(z) + \rho _0^t(z) \end{aligned}$$
- Substituting Eq. (10) into Poisson Eq. (4) and integrating along *z* gives the electric potential $\phi (z)$ and electric field distribution *E*(*z*). Using Eq. (8) the STO permittivity is updated, which combines Eq. (4) to iteratively calculate *E*(*z*) and potential $\phi (z)$.
- After obtaining the potential $\phi (z)$, we can solve the Schrödinger Eq. (3) to get eigenfunction and eigenenergy. The Fermi level can be evaluated inverting numerically the relation:
  11 $$\begin{aligned} n^m = \sum _{n,\alpha } \dfrac{\sqrt{m_\alpha ^x m_\alpha ^y}}{\pi \hbar ^2}\Theta (E_F -\varepsilon _{n\alpha } ) \end{aligned}$$
  From the knowledge of Fermi level $E_F$, the mobile charge distribution can be calculated
  12 $$\begin{aligned} \rho ^m(z) = -e \sum _{n,\alpha } \dfrac{\sqrt{m_\alpha ^x m_\alpha ^y}}{\pi \hbar ^2}\Theta (E_F -\varepsilon _{n\alpha } ) |\zeta _{n\alpha }(z)|^2 \end{aligned}$$
- A self-consistent loop is performed to obtain a converging potential $\phi (z)$ by repeating the last steps.

## Supplementary Information

Supplementary Information.

## Data Availability

The datasets used and analysed during the current study are available from the corresponding author on reasonable request.
