# Supplementary material for: Tuning of the magnetotransport properties of a spin-polarized 2D electron system using visible light
Source: Sci Rep. 2023 Jun 21;13:10050. doi: 10.1038/s41598-023-36957-w (PMC10284910; doi:10.1038/s41598-023-36957-w)
Supplement: Supplementary file 1 — Supplementary Information. [file 41598_2023_36957_MOESM1_ESM.pdf]

Tuning of the magnetotransport properties of a ferromagnetic 2DES using visible light

Supplementary information

Additional R_{xy} data

Figure S1 shows additional data extracted from the R_{xy} curves fit of a second sample. The results are in overall agreement with the data presented in the main text.

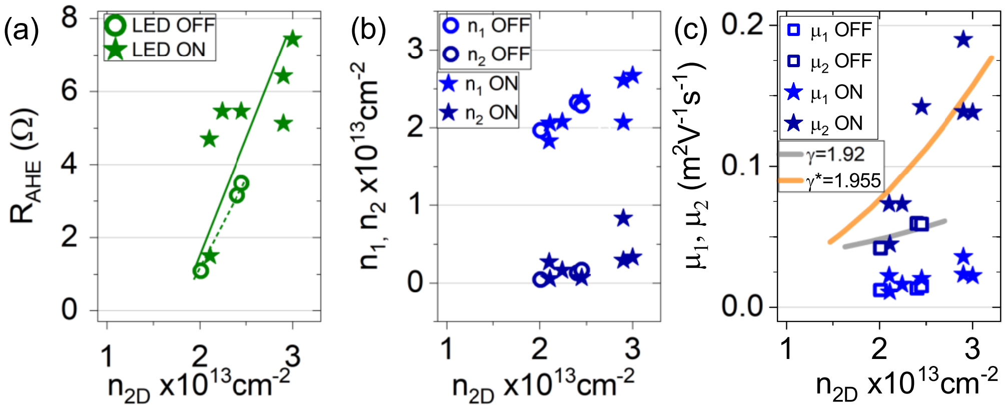

Fig. S1. Panel (a) reports the R_{AHE} values extracted from the anomalous part of the R_{xy} curves modulated using electric field effect (open dots) and light (stars). Panels (b) and (c) show the carrier concentration and mobility respectively extracted from two band fit of the Hall data. Open symbols refer to gate modulated transport while stars to light modulated one. n_1 (μ_1) and n_2 (μ_2) refer to the concentration (mobility) of carriers having d_{xy} and $d_{xz,yz}$ orbital character respectively. The full lines in panel (c) refer to the function $\mu_2 \propto n_{tot}^{\gamma}$.

Further results on Schrödinger-Poisson numerical simulations of the quantum well

The difference among top gating, back gating, and photo-doping is also depicted in the Figure S2. For these calculations, we assume that the density of mobile charges $\delta n^m = 0.9 \times 10^{13}\ cm^{-2}$ and trapped charges induced by electric field is same for back (V_{bg}=60 V) and top (V_{tg}=0.64 V) gates. Noteworthy, in the range of carrier concentration used in our experiments, simulations show that top-gating is more effective in filling carriers with d_{xy} character compared to bottom gating (Fig. S2(d)), while $d_{xz/yz}$ electrons are emerging when back gating is used (Fig. S2(b)). The $d_{xz/yz}$ band electrons extend further into STO, thus, their mobility is higher compared to the d_{xy} ones which are more confined to the interface. After illumination detraps a fraction of electrons, as mentioned, a smaller back gate (we take V_{bg}=16 V) is applied to get same density of the mobile charges δn^m (Fig. S2(c)). Comparing the Fig. S2(b) and Fig. S2(c), the band levels are slightly deeper in back gating plus illumination ($E(d_{xy}) = 86.9\,meV, 143.1\,meV$ and $E(d_{xz/yz}) = 142.9\,meV$) than what happens when only back gate is applied ($E(d_{xy}) = 92\,meV, 150.9\,meV$ and $E(d_{xz/yz}) = 155.3\,meV$). In this case, a slight deconfinement in potential causes a larger increase in mobility.

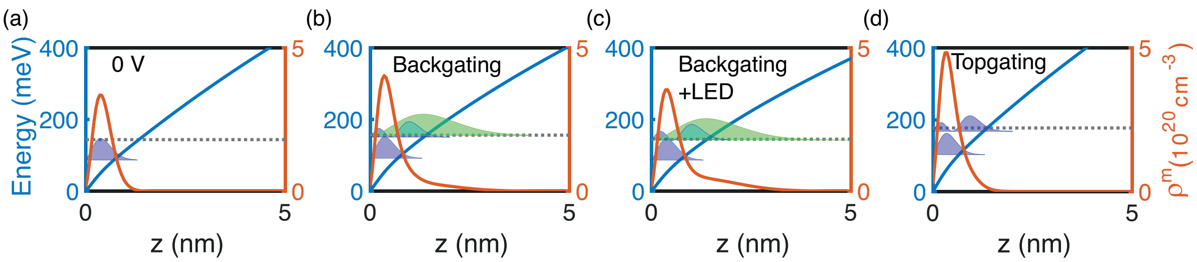

Fig. S2. Schrödinger-Poisson numerical simulations showing the spatial dependence of confining potential $V(z)$, the energies E_{xy}, $E_{xz/yz}$, the wave-function square modulus of the d_{xy} (in blue) and $d_{xz/yz}$ (in green) electrons (left axis), and carrier density distribution (right axis).

DFT+U and RESPES data on LAO/ETO/STO

FigureS3(a) reports element and spin-resolved density of states extracted from DFT+U calculations for $EuTiO_3$ layers with FM coupling. A substantial spin-polarization at -2 eV of both Eu-4f (green) and Eu-5d (light blue) states and an overlap between Ti-3d states (gray) and Eu-5d states near and above the Fermi level can be observed. Panel (b) shows angle and photon-energy integrated RESPES VB of (001) LAO(5 u.c.)/ETO(2 u.c.)/STO heterostructure. A large Eu-4f peak is visible around -2eV. Both panels are adapted from Ref. 1.

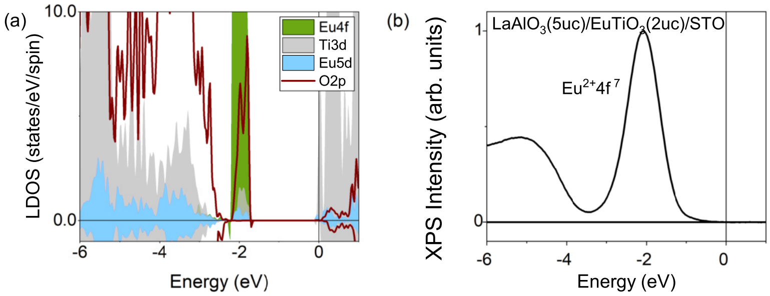

Fig. S3. Panel (a) reports element and spin-resolved density of states from DFT+U calculations for $EuTiO_3$ layers (average) with FM coupling. Eu-4f states are reported in green, Eu-5d states in light blue and Ti-3d in gray. Panel (b) shows angle and photon-energy integrated RESPES VB of (001) LAO(5 u.c.)/ETO(2 u.c.)/STO heterostructure.

References

1. Di Capua, R. *et al.* Orbital selective switching of ferromagnetism in an oxide quasi two-dimensional electron gas. *npj Quantum Mater.* **7**, 1–10 (2022).
